# Comparison of Transcriptomic Profiles of MiaPaCa-2 Pancreatic Cancer Cells Treated with Different Statins

**DOI:** 10.3390/molecules26123528

**Published:** 2021-06-09

**Authors:** Silvie Rimpelová, Michal Kolář, Hynek Strnad, Tomáš Ruml, Libor Vítek, Helena Gbelcová

**Affiliations:** 1 Department of Biochemistry and Microbiology, University of Chemistry and Technology Prague, Technická 3, 166 28 Prague, Czech Republic; tomas.ruml@vscht.cz; 2 Laboratory of Genomics and Bioinformatics, Institute of Molecular Genetics, Czech Academy of Sciences, Vídeňská 1083, 142 20 Prague, Czech Republic; michal.kolar@img.cas.cz (M.K.); hynek.strnad@img.cas.cz (H.S.); 3 Institute of Medical Biochemistry and Laboratory Diagnostics, and 4th Department of Internal Medicine, 1st Faculty of Medicine, Charles University, 128 08 Prague, Czech Republic; vitek@cesnet.cz; 4 Institute of Medical Biology, Genetics and Clinical Genetics, Faculty of Medicine, Comenius University, Špitálska 24, 813 72 Bratislava, Slovakia

**Keywords:** statins, pancreatic cancer, DNA microarray, pitavastatin, cerivastatin, simvastatin, fluvastatin, atorvastatin, pravastatin, HMG-CoA reductase inhibitors

## Abstract

Statins have been widely used for the treatment of hypercholesterolemia due to their ability to inhibit HMG-CoA reductase, the rate-limiting enzyme of de novo cholesterol synthesis, via the so-called mevalonate pathway. However, their inhibitory action also causes depletion of downstream intermediates of the pathway, resulting in the pleiotropic effects of statins, including the beneficial impact in the treatment of cancer. In our study, we compared the effect of all eight existing statins on the expression of genes, the products of which are implicated in cancer inhibition and suggested the molecular mechanisms of their action in epigenetic and posttranslational regulation, and in cell-cycle arrest, death, migration, or invasion of the cancer cells.

## 1. Introduction

**Figure 1:**
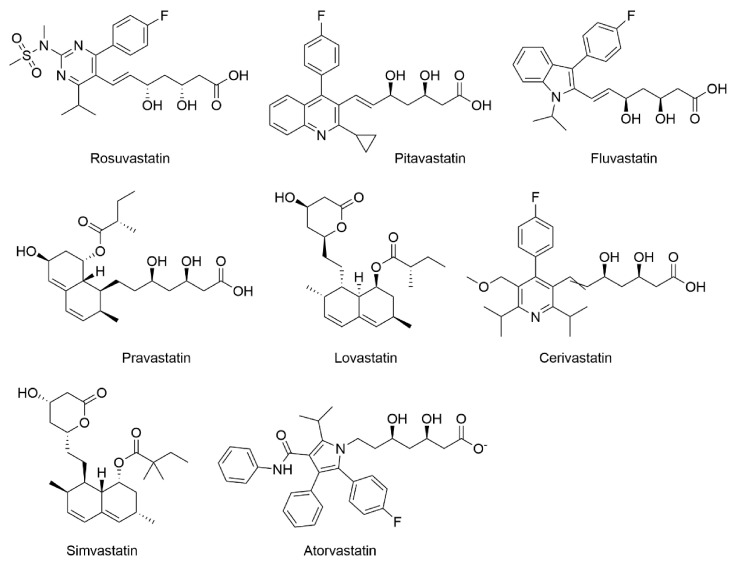
Chemical structures of statins.

Statins (Figure 1) are the inhibitors of *de novo* cholesterol synthesis in the cell due to their ability to competitively inhibit the β-hydroxy-β-methylglutaryl coenzyme A (HMG-CoA) reductase. They represent the most prescribed drugs in the treatment of cardiovascular diseases [1]. Currently, there are eight existing statins, namely atorvastatin, cerivastatin, fluvastatin, lovastatin, pitavastatin, pravastatin, rosuvastatin, and simvastatin [2]. Although all statins have the same mechanism of hypolipidemic action, they differ in their chemical structure, physico-chemical properties, pharmacokinetic effects, and effects on lipid profile [3]. The inhibition of HMG-CoA conversion to mevalonate caused by HMG-CoA reductase inhibition results in the depletion of downstream intermediates of the mevalonate pathway.

Mevalonate is the precursor of, i.e., farnesyl pyrophosphate (FPP), geranylgeranyl pyrophosphate (GGPP), isopentenyl adenine, dolichol, and the polyisoprenoid side chains of heme A and ubiquinone, which are essential molecules that play a vital role in almost any cell process [3]. From these, FPP and GGPP play an important role in the posttranslational modification of cellular proteins involved in cell division and differentiation, gene expression, cytoskeleton formation, intracellular protein and lipid transport, and defense against pathogens [4]. Another intermediate in the mevalonate pathway, isopentenyl adenine, is essential for proper tRNA function and protein synthesis. Dolichol acts as an important scavenger of free radicals in cell membranes, and ubiquinone is involved in mitochondrial respiration and inhibition of lipid peroxidation. A decrease in the intracellular level of ubiquinone leads to mitochondrial damage, oxidative stress, and cell damage. The involvement of the aforementioned mevalonate pathway intermediates in various cell processes explains the pleiotropic activity exhibited by statins, in addition to their hypolipidemic effect.

Based on this, it is clear that statins significantly affect basic physiological processes of cells and organs that also are connected with oncogenesis [5]. The results of lipid-lowering therapy in animals initially indicated an increased risk of carcinogenesis [6]. However, the dose of statins administered in that study was very high and not applicable for humans [7]. The results of studies on tissue-specific cancer development in individuals on statins therapy (as hypolipidemic drugs) have been controversial. An increased incidence of breast cancer after statin administration was observed in one study [8], but another study did not confirm these findings [9]. Contrary to that, a reduced incidence of melanomas has been reported after statin administration [9]. A similar beneficial effect of statins was observed in connection with reduced incidence of colon carcinomas [10]. However, most of these studies were not originally designed to investigate the relationship between statin intake and cancer development, but rather between statins and cardiovascular diseases. Indeed, some reports have shown a statistically significantly lower incidence of cancer in patients receiving statin therapy, despite relatively short follow-up times and inappropriate patient selection [11,12,13,14,15,16]. Moreover, extensive studies conducted on 500,000 U.S. war veterans have shown that statin use is associated with a two- to fivefold lower incidence of lung [17], breast [18], and prostate cancers [19]. Based on other meta-analyses, statins appear to be particularly effective in the chemoprevention of colorectal cancer [10,20]. While there have been several *in vitro* and *in vivo* studies directly targeting and confirming the marked effect of statins on the growth of a wide variety of tumor types, such as hepatocellular [21], lung [22], and colorectal carcinoma [23], clinical trials targeting statin efficacy in cancer treatment have been very rare, so far. One of them showed that patients suffering from hepatocellular carcinoma exhibited statistically significantly longer survival rates for pravastatin therapy [24] than untreated. On the other hand, beneficial effects of lovastatin therapy on the survival rate of patients suffering from glioblastoma multiforme or advanced gastric adenocarcinoma have not been observed [25,26]. The findings of the meta-analyses suggested an association between pravastatin treatment and cancer in elderly patients [27] but did not support the potential role of statins in the prevention of hematological malignancies [28] or the hypothesis that statins reduce the risk of pancreatic cancer development [29].

The setting of the experimental conditions of the present study was based on the results of our previous published studies [30,31], in which we reported on the differences in the individual anticancer potential of individual statins in cells derived from pancreatic carcinoma. Moreover, we were inspired by the aforementioned findings, as well as recent reviews shedding light on statins as cancer inhibitors with several possible mechanisms of action [32,33,34,35]. Therefore, using DNA microarray analysis, we sought after statin-induced changes in the expression of genes, the products of which are connected with tumorigenesis, inhibition of tumor growth, or metastasis. We focused on the pancreatic cancer cells because, in the very recent meta-analysis of 26 studies containing more than 3 million participants and 170,000 pancreatic cancer patients, statin therapy was shown to significantly reduce the risk of pancreatic cancer [36].

## 2. Results

From eight existing statins, only six (cerivastatin, pitavastatin, simvastatin, lovastatin, fluvastatin, and atorvastatin) significantly affected the gene expression in the MiaPaCa-2 pancreatic cancer cell line after 24 h of treatment with 12 µM statin concentration. The concentration was selected based on our previous studies [30,31], in which we determined the IC_50_ of individual statins in cells from pancreatic carcinoma after 24, 48, and 72 h. The value 12 µM corresponds to the IC_50_ of simvastatin in MiaPaCa-2 cells after 24 h of treatment. Other statins were compared at the same concentration since simvastatin was at the time of analysis one of the most potent clinically used statins (cerivastatin had been withdrawn from the market at the time of analysis).

### 2.1. Effect of Statins on Lipid Metabolism and Synthesis of Steroids

Lipids, particularly cholesterol, and their derivatives play several roles in tumorigenesis and cancer cell metabolism [37,38]. In contrast to noncancerous cells, malignant cells exhibit enhanced *de novo* synthesis of fatty acids, which serve as substrates for β-oxidation, or conversion to triglycerides for storage of phospholipids for membrane formation.

**Figure 2:**
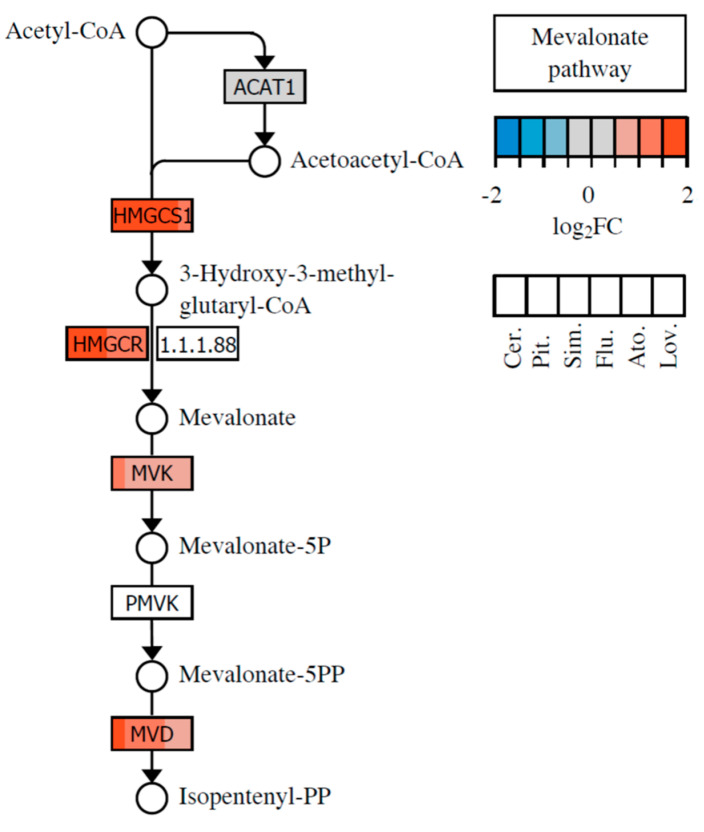
Mevalonate pathway and the changes induced by statin treatment in the expression of the genes that code for enzymes of the pathway. The color fill of the nodes indicates the base-2 logarithm of the fold change in gene expression upon treatment by a statin. Different statins are shown in the distinct position of the node as indicated in the bottom key.

DNA microarray analysis showed that out of the eight statins evaluated, six effective ones significantly affected steroid biosynthesis in MiaPaCa-2 cells. They upregulated the gene encoding HMG-CoA reductase, representing the key target of statins. In addition, they even more markedly triggered upregulation of the gene encoding HMG-CoA synthase, catalyzing the synthesis of HMG-CoA, the substrate for the aforementioned enzyme covalently inhibited by statins. The inhibition of the mevalonate pathway resulted in a switch from cholesterol synthesis to the triacylglyceroles and phospholipid synthesis via the Kennedy pathway. Genes encoding enzymes of the Kennedy pathway were directly upregulated by the action of only the most effective statins; i.e., cerivastatin, pitavastatin, and simvastatin (Table 1). However, in terms of knowledge of the lipid metabolism of tumors, this observation indicated a procarcinogenic effect rather than an antitumor effect of statins. The changes in the expression of the genes involved in the mevalonate pathway are shown in Figure 2.

On the other hand, statins are responsible for a decrease in the cholesterol content in lipid rafts by inhibiting the mevalonate pathway and, thus, induce apoptosis via inhibition of the Akt signaling pathway [39]. Interestingly, in our study, the Akt signaling pathway was not affected by the evaluated statins at 12 µM after 24 h treatment of MiaPaCa-2 cells.

In tumors with deregulated Hedgehog signaling, the depletion of cholesterol results in impairment of Hedgehog signal transduction and inhibition of cancer cell growth [40]. Cholesterol itself serves as a substrate for the post-translational modification of Hedgehog ligands, which is required for their proper trafficking [41]. This means that all statins should indirectly inhibit the Hedgehog signaling by inhibiting the cholesterol synthesis. Moreover, we found that pitavastatin and cerivastatin significantly upregulated the *GAS1* (growth arrest-specific gene 1) (data not shown), the product of which positively regulates the Hedgehog signaling [42,43]. The possible explanation for *GAS1* gene upregulation after statin treatment was the effort of affected cells to reactivate the statins-suppressed Hedgehog signaling.

Finally, to maintain cancer cell proliferation, the increased activation of SREBPs (sterol regulatory element-binding proteins) is required [44]; therefore, SREBP inhibitors are used in molecular-targeted cancer therapies [45,46]. SREBPs are transcription factors that regulate the expression of genes required for the synthesis of fatty acids, triglycerides, and cholesterol. Intracellular cholesterol level is controlled by SREBP-1 and SREBP-2. These transcription factors upregulate the synthesis of enzymes involved in the sterol biosynthesis upon binding to specific sterol regulatory element DNA sequences. Sterols in turn inhibit the cleavage of SREBPs, and therefore, synthesis of additional sterols is reduced via a negative feedback loop. Statin-mediated inhibition of sterol synthesis was reported to activate the SREBPs in many tumor types [45]. In our experiment, statins at a concentration (12 µM) sufficient to induce a more or less intensive antiproliferative effect on MiaPaCa-2 pancreatic cancer cells (IC_50_: cerivastatin 10 µM, simvastatin 12 µM, lovastatin 13 µM, pitavastatin 20 µM, fluvastatin 26 µM, atorvastatin 27 µM, pravastatin 29 µM, rosuvastatin 36 µM) did not significantly affect the expression of genes encoding SREBPs (data not shown).

### 2.2. Statins in the Role of Epigenetic Regulators

**Figure 3:**
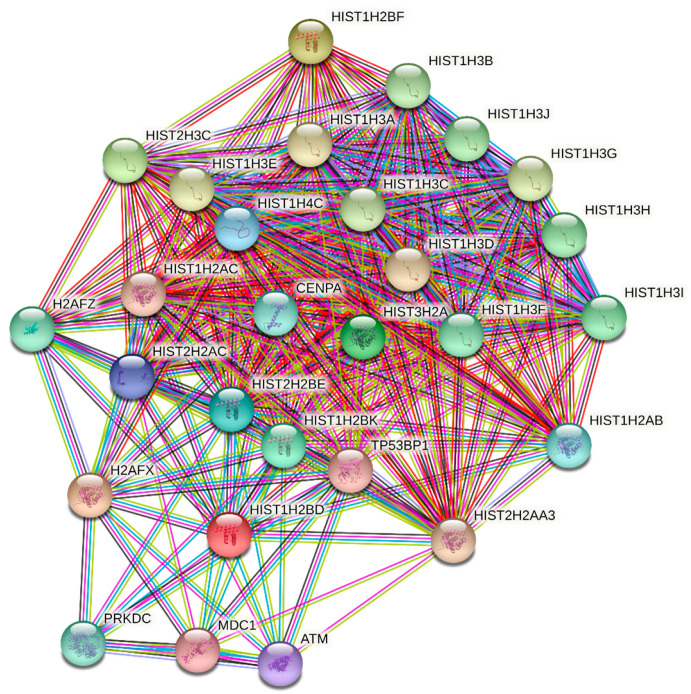
STRING enrichment analysis of epigenetic regulators significantly affected by statin treatment (mainly by cerivastatin and pitavastatin) of MiaPaCa-2 cells at 12 µM concentration for 24 h. Individual nodes represent affected gene products and their interactions. Input nodes represent the genes listed in Table 2 (*HIST1H4C, HIST1H2BF, HIST2H3C, H2AFX, HIST2H2BE, HIST1H2BD, HIST1H2BK,* and *HIST3H2A*). The evidence view of the association network was generated according to the known and predicted interactions in *Homo sapiens*. Known interactions: turquoise—from curated databases; violet—experimentally determined. Predicted interactions: green—gene neighborhood; red—gene fusions; blue—gene co-occurrence. Others: grass green—text-mining; black—co-expression; light blue—protein homology. A complete description of the nodes is given in Supplementary Table S1.

Recently, it was described that acetyl-CoA metabolism supports multistep pancreatic tumorigenesis. In pancreatic adenocarcinoma, both upregulated gene expression of the mevalonate pathway and histone acetylation was detected [47]. Dysregulation between activities of histone acetyltransferases (HATs) and histone deacetylases (HDACs) is frequent in human tumors [48]. The expression of genes encoding HATs or HDACs was not altered by statins in our experiments (data not shown); however, genes encoding histone H4 were downregulated, in contrast to genes for histone H2B that were upregulated by cerivastatin, pitavastatin, and simvastatin (Table 2). STRING enrichment analysis of epigenetic regulators significantly affected by statin treatment is shown in Figure 3.

### 2.3. Statins and Their Potential Role as Posttranslational Regulators

**Figure 4:**
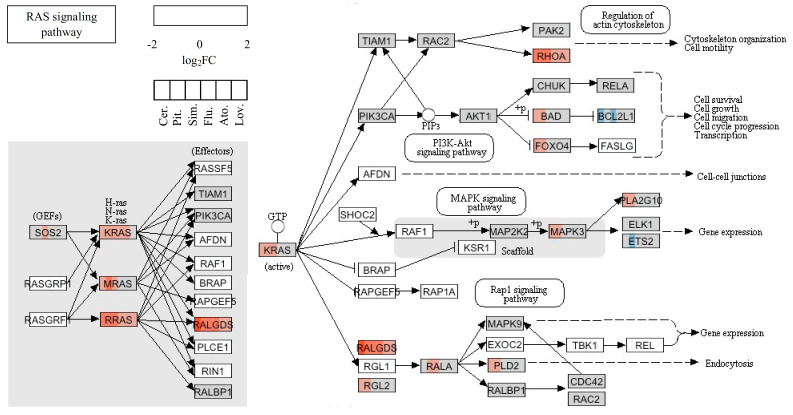
RAS signaling pathway and the changes induced by statin treatment in the expression of the genes that code for members of the pathway. The color fill of the nodes indicates the base-2 logarithm of the fold change in gene expression upon treatment by a statin. Different statins are shown in the distinct position of the node as indicated in the key.

Statins deplete the cellular pool of isoprene precursors, thereby having an impact on protein prenylation; i.e., farnesylation and geranylgeranylation. Among prenylated proteins, the low-molecular-weight guanosine triphosphate-binding proteins Ras and Ras-related growth modulators were monitored (Table 3). The overall effect on the RAS signaling pathway is shown in Figure 4.

*RHOB*, the most dramatically upregulated gene by statins (Table 3), belongs to the Rho protein family of proteins regulating diverse cellular processes, including cytoskeletal organization, gene transcription, cell cycle progression, and cytokinesis [49,50]. While most Rho proteins have been shown to have positive roles in proliferation and malignant transformation, RhoB rather appears to act as a negative regulator of these processes [51,52]. R-Ras promotes the formation of focal adhesions, cell spreading, and activation of integrins [53]. Cerivastatin, pitavastatin, simvastatin, and fluvastatin also increased the expression of the *KRAS* gene, the most frequently mutated gene in pancreatic cancer, and frequently mutated in cancer in general. As the K-Ras is the protein related to many functional pathways, such as the mitogen-activated protein kinase (MAPK) signaling pathway, the receptor tyrosine-protein kinase (ErbB) signaling pathway, dorso-ventral axis formation, axon guidance, the vascular endothelial growth factor (VEGF) signaling pathway, tight junctions, gap junctions, natural killer cell-mediated cytotoxicity, the T-cell receptor signaling pathway, the B-cell receptor signaling pathway, the Fc epsilon RI signaling pathway, long-term potentiation, long-term depression, regulation of actin cytoskeleton, the insulin signaling pathway, and the gonadotropin-releasing hormone (GnRH) signaling pathway, the pleiotropic effect of statins is comprehensible. Moreover, the *KRAS* gene upregulation after statin treatment adverts to the unavailability of K-Ras protein for the cell signaling without its correct posttranslational modification due to the mevalonate pathway inhibition. Similarly, this mechanism could also explain the upregulation of other Ras and Ras-related proteins induced by treatment with simvastatin. Genes encoding guanine nucleotide exchange factor (GEF), GTPase-activating proteins (GAP), or guanosine nucleotide dissociation inhibitor (GDI) of Ras and Ras-related proteins were not significantly up- or downregulated by statins.

### 2.4. Statins’ Effect on Cell Cycle and Cell Death

**Figure 5:**
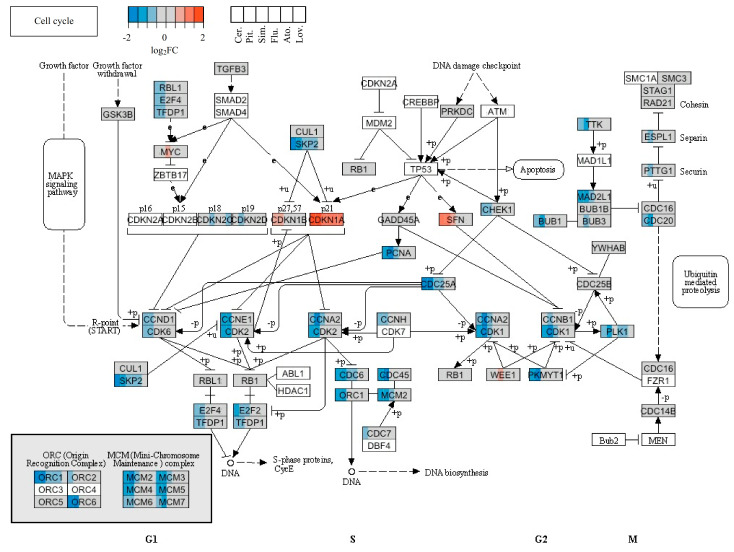
Cell-cycle signalization and the changes induced by statin treatment in the expression of the genes that code for members of the circuit. The color fill of the nodes indicates the base-2 logarithm of the fold change in gene expression upon treatment by a statin. Different statins are shown in the distinct position of the node as indicated in the key.

The effect of statins on the expression of genes related to the cell cycle and cell death is of special interest concerning their cancerostatic capability. The expression levels of genes associated with DNA replication were significantly affected only by pitavastatin, cerivastatin, and simvastatin, and they were mostly downregulated (Table 4). The changes in the cell-cycle circuit are shown in Figure 5.

The origin recognition complex (ORC) is a highly conserved six-subunit protein complex essential for the initiation of DNA replication in eukaryotic cells. The ORC binds specifically to origins of replication and serves as a platform for the assembly of additional initiation factors such as minichromosome maintenance (MCM) proteins. ORC1L is the largest subunit of the ORC complex. While the levels of other ORC subunits are stable throughout the whole cell cycle, the level of ORC1L changes in various phases of the cell cycle. These changes are controlled by ubiquitin-mediated proteolysis after the initiation of DNA replication [54]. From this, it seems that statins blocked the progression of the cell cycle through the S phase. From the quantum of genes encoding proteins involved in the cell-cycle regulation, such as cyclins, cyclin-dependent kinases (CDK), and cell-cycle negative regulators, only the expression of those denoted in Table 4 were affected by statins. CDC2 (CDK1) is the catalytic subunit of a highly conserved protein kinase complex known as the M-phase promoting factor (MPF), which is essential for the G1/S and G2/M phase transitions in the cell cycle of eukaryotic cells. Mitotic cyclins stably associate with this protein and function as regulatory subunits. The kinase activity of this protein is controlled by cyclin accumulation and degradation through the cell cycle [55].

M-phase inducer phosphatase 2 (CDC25B), a member of the cell division control protein 25 (CDC25) family of phosphatases, activates the cyclin-dependent kinase (CDC2) and is required for entry into the mitosis [56]. Whereas the *CDC2* gene was downregulated by simvastatin treatment of MiaPaCa-2 cells, the *CDC25B* gene was upregulated (Table 4). Some other genes encoding proteins related to the S phase (SKP2, E2F2) were downregulated by cerivastatin and pitavastatin. The SKP2 (S-phase kinase-associated protein 2) is an essential element of the cyclin A/CDK2 S-phase kinase [57]. E2F2 is a member of the E2F family of transcription factors and plays a crucial role in the control of the cell cycle. Expression of the S-phase genes is not activated when E2F is repressed [58].

**Figure 6:**
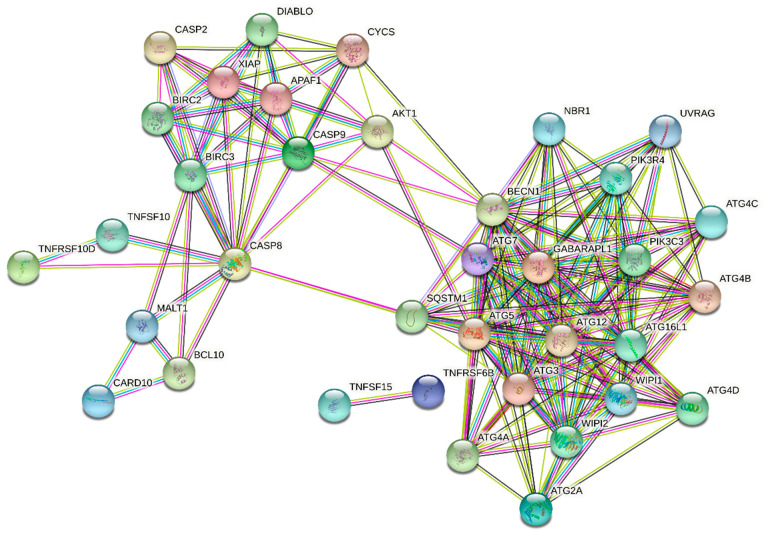
STRING enrichment analysis of products of genes involved in cell death significantly affected by statin treatment (mainly by cerivastatin and pitavastatin) of MiaPaCa-2 cells at 12 µM concentration for 24 h. Individual nodes represent affected gene products and their interactions. Input nodes were based on the genes listed in Table 5 (*TNFRSF10D, SLC6A12, DRAM, CASP9, GABARAPL1, ATG2A, TNFAIP1, CARD10,* and *TNFRSF6B*). A complete description of the nodes is given in Supplementary Table S1. See Figure 3’s caption for color coding of the edges.

In our experimental setup, one of the genes most affected by statin treatment was *TNFRSF10D* (Table 5). Its product, the tumor necrosis factor receptor superfamily member 10D precursor (known also as TNF-related apoptosis-inducing ligand receptor 4, or TRAIL receptor 4) is a member of the TNF-receptor superfamily, which is closely connected with apoptosis. TNFRSF10D has been shown to play an inhibitory role in TRAIL-induced cell apoptosis [59]. Upregulation of another gene, *GABARAPL* (also known as early estrogen-regulated protein; Table 5) suggests that statin treatment may induce cell death by autophagy [53]. STRING enrichment analysis of products of genes involved in cell death significantly affected by statin treatment is shown in Figure 6.

### 2.5. Statins’ Effect on Migration and Invasion of Cancer Cells

**Figure 7:**
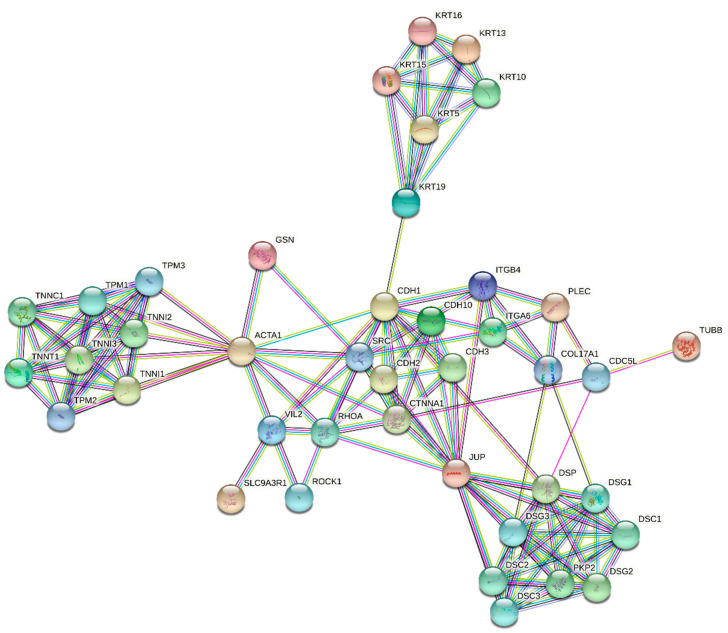
STRING enrichment analysis of products of genes involved in cytoskeleton maintenance significantly affected by statin treatment (mainly by cerivastatin, pitavastatin, and simvastatin) of MiaPaCa-2 cells at 12 µM concentration for 24 h. Individual nodes represent affected gene products and their interactions. Input nodes were based on the genes listed in Table 6 (*LOC400578, KRT16, MGC102966, KRT15, KLHL24, JUP, TUBB1, KRT13, KRT19, GSN, KIF1A, TNNT1, ITGB4, VIL2, NAV1, GSN, CDH10,* and *SYNM*). A complete description of the nodes is given in Supplementary Table S1. See Figure 3’s caption for color coding of the edges.

Since statins are known to affect migration and invasion of various cancer cells [60,61,62,63,64], which is also in accordance with our preliminary unpublished data, we also concentrated on the analyses of changes in the transcription of genes involved in cell migration and cytoskeleton architecture. The pancreatic cancer cells used in our study were firmly attached to the cultivation surface. However, the statin treatment induced a change in the cell shape and facilitated their detachment (data not shown). This is consistent with the effect of statins on the expression of the genes encoding the cytoskeletal proteins (Table 6). STRING enrichment analysis of products of genes involved in cytoskeleton maintenance significantly affected by statin treatment is shown in Figure 7.

Intermediate filaments (IF) of the cytoplasmic cytoskeleton are composed of keratins that are the major structural proteins of epithelial cells. They interact with desmosomes and hemidesmosomes, by which they assist in cell–cell adhesion [65].

TNNT1 is a subunit of troponin, which is a regulatory complex located on the thin filament of the sarcomere. This complex regulates striated muscle contraction in response to fluctuations in intracellular calcium concentration [66]. Gelsolin is an actin-binding protein that is a key regulator of actin filament assembly and disassembly. Among the lipid-binding actin regulatory proteins, gelsolin is one of the few that exhibit preferential binding toward polyphosphoinositide. The activity of gelsolin is stimulated by calcium ions (Ca^2+^) [67]. Kinesins belong to a class of motor proteins found in eukaryotic cells. Kinesins move along microtubule cables powered by the dephosphorylation of adenosine triphosphate (ATP). The active movement of kinesins supports several cellular functions, including mitosis, meiosis, and transport of cargo [68]. Catenins are proteins found in complexes with cadherin cell adhesion molecules of animal cells. Junction plakoglobin (catenin gamma) was originally identified as a component of desmosomes (a cell structure specialized for cell-to-cell adhesion) [69]. Ezrin is the cytoplasmic peripheral membrane protein that serves as an intermediate between the plasma membrane and the actin cytoskeleton. This protein plays a key role in cell-surface structure adhesion, migration, and organization, and it has been implicated in various human cancers [70]. CDH10 is an integral membrane protein that mediates calcium-dependent cell–cell adhesion [71].

## 3. Materials and Methods

### 3.1. DNA Microarray Analysis

Description of an experimental method for the studying of the effects of individual statins on the gene-expression profile of MiaPaCa-2 cells was reported by Gbelcová et al. [30]. We compared the effect of statins to simvastatin because it was chosen as the most effective clinically used statin tested *in vitro* in our previous study. Briefly, pure forms (≥98 %) of all commercially available statins were used: atorvastatin, lovastatin, simvastatin, fluvastatin, cerivastatin, pravastatin, rosuvastatin, and pitavastatin (LKT Laboratories, USA). Statins were dissolved in methanol and tested in 12 μM concentrations, representing the IC_50_ value for simvastatin after 24 h of treatment of MiaPaCa-2 cancer cells. Human pancreatic cancer cells MiaPaCa-2 (ATCC, Manassas, VA) were cultured in DMEM medium (Sigma Aldrich, Germany) supplemented with 10 % fetal bovine serum. Illumina HumanWG-6_V3 chips (Illumina, USA) were used for the microarray analysis following the standard protocol. Annotation of differentially expressed transcripts was done with R/BioConductor packages [72] against the Ensembl database (version 47) [73]. The transcripts with a false discovery rate smaller than 0.05 and fold change smaller than 0.5 or greater than 2 were reported and used in the downstream analysis. Changes in the gene expression were visualized using the Pathview package [74] on the pathways provided by the KEGG database [75].

### 3.2. RT-qPCR Analysis

Validation of the selected gene expression changes was performed using quantitative RT-PCR. The amount of 1 × 10^8^ cells was washed with phosphate-buffered saline and lysed using the RLT buffer (Qiagen, Hilden, Germany) supplemented with β-mercaptoethanol. The total RNA was extracted by the RNeasy Micro kit (Qiagen) according to the manufacturer’s protocol. All extracts were treated with DNase I (Qiagen) to remove contaminating genomic DNA. The quality and quantity of the RNA were evaluated with an Agilent 2100 Bioanalyzer instrument using the RNA 6000 Nano kit (both Agilent Technologies, Santa Clara, CA, USA). Expression levels of the mRNA were determined using a two-step RT-qPCR method. First, cDNA was reverse-transcribed from 1 μg of total RNA in a final reaction volume of 20 μL using a QuantiTect reverse transcription kit (Qiagen) according to the manufacturer’s instructions. Then, the cDNA concentration was quantified using a LightCycler 480 instrument in LightCycler 480 SYBR Green I master mix (both Roche Applied Sciences, Penzberg, Germany) with a custom primer mix (see Supplementary Table S1 for primer sequences). For each condition, two biological replicates were analyzed (three for the control group).

Data analysis was performed using the delta–delta Cq method [76] within the R statistical environment [77]. Cq values were truncated at the value of 40. Three housekeeping genes (RPS9, TBP, and GAPDH) were used as reference genes for RNA quantity normalization using the geNorm algorithm [78]. Relative expression levels were computed assuming the perfect efficiency of the PCR.

### 3.3. STRING Analysis

A functional association network of known and predicted functional partners or selected genes identified as significantly changed in the microarray analysis in MiaPaCa-2 cells treated with 12 µM concentration of statins for 24 h was created using STRING database 11.0 [79]. STRING is an interaction network database for functional enrichment analysis of protein–protein interaction networks. Input nodes were chosen based on the most important hits identified by the microarray analysis and depicted in Figure 3, Figure 6, and Figure 7. The evidence view diagrams were generated by STRING to illustrate the known protein–protein interactions of all connected nodes. The view of the association network was done for *Homo sapiens* according to the known and predicted from curated databases, experimentally determined, gene neighborhood, gene fusions, gene co-occurrence, text-mining, co-expression, and protein homology. The confidence score was set to high, equal to 0.700, with a maximum of 30 interactions. Disconnected nodes in the networks were hidden.

## 4. Discussion and Conclusions

Statins have been intensively studied drugs based on their deep impact on the human organism caused, in particular by their cholesterol-lowering activity. However, their effects also include remarkable and potentially clinically relevant antitumor effects.

Although lipids are not genetically encoded, the genome changes can reflect cholesterol homeostasis indirectly. The changes in gene expression following statin treatment studied by microarray technology have been reported since the year 2000. The interpretation of our microarray analysis indicates a significant correlation between our and previously reported results. On the other hand, many differences are resulting from distinct experimental conditions due to the type of statin used, its concentration and duration of its activity, the experimental model (the type of a cell line or organism), etc.

Previously, we have demonstrated substantial differences in cancer cell antiproliferative effects of all commercially available statins in an experimental model of human pancreatic cancer [31]. Various statins exhibited significantly different inhibitory efficacy, and we have also observed notable differences in statin sensitivity between pancreatic cancer cell lines, concerning the level of differentiation and harboring of G12C-activating mutation in the *KRAS* gene. Cerivastatin, pitavastatin, and simvastatin were the most effective, whereas rosuvastatin and pravastatin were the least effective, and their effectiveness correlated with the properties of the cell lines tested (the level of differentiation, and presence of G12C *KRAS* mutation) [31]. In the present study, we observed that depletion of farnesylated K-Ras protein caused by the three most effective statins led to significant upregulation of gene-encoding K-Ras.

The pleiotropic effect of statins is comprehensible, since K-Ras is related to many functional pathways, such as the MAPK signaling pathway, the ErbB signaling pathway, dorso-ventral axis formation, axon guidance, the VEGF signaling pathway, tight junction, gap junction, natural killer cell-mediated cytotoxicity, the T-cell receptor signaling pathway, the B-cell receptor signaling pathway, the Fc epsilon RI signaling pathway, long-term potentiation, long-term depression, regulation of actin cytoskeleton, the insulin signaling pathway, and the GnRH signaling pathway. Moreover, the upregulation of KRAS after the statin treatment seemed to be a result of the unavailability of farnesylated K-Ras protein for cell signaling due to the mevalonate pathway inhibition. Similarly, this mechanism could also explain the upregulation of other Ras and Ras-related proteins induced by statin treatment (Table 3). However, the products of the mevalonate pathway are required not only for post-translational modifications of many proteins, but also for regulation of the transcription of many proteins, including proteins of the cytoskeleton. For example, expression of keratin 13 is known to be regulated by nuclear receptor ligands such as retinoids, 1α, 25-dihydroxy vitamin D3, or estrogen [65]. Except for the metabolism of lipids (biosynthesis of steroids and sphingolipid metabolism), the inhibition of the mevalonate pathway by statins directly affected the energy metabolism due to ubiquinone depletion, as ubiquinone is involved in mitochondrial respiration [80].

Genetic heterogeneity is an important factor that affects the sensitivity of particular cancer cells to the antiproliferative/proapoptotic effects of statins. Despite the level of cancer cell differentiation, pancreatic cancer cell lines harboring activating K-Ras mutation were more sensitive to the antiproliferative effect of statins than cells harboring wild-type K-Ras [31]. Similarly, Wong et al. demonstrated that only 8 of 17 multiple myeloma cell lines evaluated were sensitive to lovastatin-induced apoptosis, while resistant cell lines had different genetic profiles [81].

The first effect of statins, specifically simvastatin, studied by microarray analysis was focused on actomyosin contraction, gap formation, and barrier dysfunction produced by thrombin. The experiment was performed in human pulmonary artery endothelial cells (EC) treated with 5 µM simvastatin for 24 h. Several genes related to thrombin-mediated cytoskeletal dynamics and barrier regulation, including caldesmon and the thrombin receptor PAR-1, were dramatically downregulated. In addition, ITGB4, a protein known to be involved in cell–cell adhesion, was dramatically upregulated. *RhoA* and *RhoC* genes were also upregulated similarly to Rac1 and specific GEFs, potential regulators of preferential Rho GTPase activity. In contrast, the RhoGDP dissociation inhibitor was downregulated, which was interpreted as a compensatory response [82]. Consistent with these data, our results also indicated that statins directly affected the expression of specific genes related to the Rho GTPase signaling and cytoskeletal regulation. However, from all the Rho family members, only *RhoB* was significantly upregulated by all effective statins (pravastatin and rosuvastatin were not effective in our microarray study), and *RhoA* was significantly upregulated only by the most effective statins (cerivastatin, pitavastatin, and simvastatin). No changes were observed in the expression of genes encoding specific GEFs. Also, downregulation of caldesmon or the thrombin receptor PAR-1 was not observed in our study. This could vary in different types of cell lines evaluated.

Interestingly, a gene encoding the integrin beta 4 subunit of a receptor for the laminins; i.e., *Itgb4*, was the most significantly upregulated gene (fold change—7.57) of all the tested genes [83]. The reason for the enhanced transcription of a gene involved in the biology of invasive carcinoma is unclear. Another notable finding is that no changes were observed in the expression of the *RhoB* gene after treatment of EC with 5 µM simvastatin [84], whereas in our study, the *RhoB* gene was the more affected gene compared to the Itgb4 gene after treatment with all effective statins.

In the other study, the DNA microarrays were used to identify gene expression patterns in the cerebral cortex of mice treated with simvastatin (50 mg/kg b.wt.) by daily oral doses for 21 days. The maximum average concentration of simvastatin was determined as 600 pmol/g in brains. This study revealed the influence of simvastatin on the expression of several genes involved in cell growth and signaling. *C-fos*, *c–myc*, and *Bcl-2* were of particular interest due to the linkage of simvastatin with cell growth and apoptosis. Simvastatin significantly reduced the expression of the proto-oncogene *c-fos*. On the other hand, it significantly increased the expression of the oncogene *c-myc* and antiapoptotic gene *Bcl-2* [84,85]. In contrast to these data, neither the expression of previously mentioned genes nor of other genes attributed to apoptotic cell death (e.g., genes encoding death receptors or caspases, the only exception to which was caspase 9, which was upregulated by cerivastatin) were affected by statins in our microarray analysis. However, as demonstrated by the analysis of the most affected functional pathways, statins affected some processes related to DNA repair, such as base excision repair or mismatch repair [30]. It is known that failure of these processes could be followed by programmed necrosis [86]. Finally, the upregulation of the *GABARAPL* gene related to autophagy was observed (Table 5). Autophagy does not always result in programmed cell death, but it represents the important mechanism for catabolic production of ATP during nutrient stress, and also plays an important role in the turnover of proteins and organelles under nutrient-replete conditions [87]. The statin-treated cells were in nutrient stress due to inhibition of the mevalonate pathway. From many end products of this pathway, ubiquinone is required in a process of ATP formation during oxidative phosphorylation. Moreover, oxidative phosphorylation is also reported as one of the significantly affected functional pathways by statins [30]. Interestingly, it was published that ubiquitinated hydrophobic proteins that are prone to aggregation are kept on the surface of lipid droplets and subjected to autophagy, as well as proteasomal degradation [88].

The cell-cycle arrest represents another frequently discussed event associated with statins. Many reports describe the effect of individual statins on the expression of cell-cycle-related genes. For example, changes in the expression of genes related to the cell cycle in chronic myelogenous leukemia cells K562 were described. Fifteen downregulated and 9 upregulated cell-cycle-related genes were observed in the presence of 20 µM simvastatin for 48 h. The results of flow cytometry showed that the cell cycle was arrested in the G1 phase [89]. Assmus et al. performed a microarray analysis of about 12,000 genes in endothelial progenitor cells (a primary cell line) treated with 0.1 µM atorvastatin for 10 h. The expression of cyclins and proliferating cell nuclear antigen (PCNA) was increased after atorvastatin treatment. Moreover, the expression of the cell-cycle inhibitory protein p27 was reduced [90]. In the next study, downregulation of cyclin D1, PCNA, and c-myc, and upregulation of p21 and p19, were provided by the treatment of human breast cancer cells with cerivastatin [91].

In our microarray analysis, the expression of genes encoding cyclin D1 was affected only by cerivastatin, cyclin A2 by pitavastatin, and cyclin E2 by both mentioned statins, similar to the *PCNA* gene. Other statins did not affect the expression of genes encoding cyclins or PCNA. The expression of the *p21* gene was increased by all effective statins. Moreover, the genes associated with DNA replication, such as *ORC1L*, *MCM2*, or *MCM3,* were downregulated by cerivastatin and pitavastatin (Table 4). This suggests that statins blocked the progress of the cell cycle through the S phase of MiaPaCa-2 cells. The downregulation of the genes encoding histone H4 and upregulation of the gene encoding histone H2B by the three most effective statins (cerivastatin, pitavastatin, and simvastatin) also was very interesting. The effect of simvastatin on expression of the gene encoding histone was also observed in a report by Johnson-Anuna et al., in which the expression level of the gene encoding the linker histone H1.2 was increased after simvastatin therapy [84]. Likewise, other proteins related to the S phase (SKP2, E2F2) or M phase (CDC2, CDC25B) were downregulated by cerivastatin and pitavastatin treatment of MiaPaCa-2 cells in our study, indicating that except for the G1 phase, the most effective statins blocked the cell-cycle entry into the M phase. This was not surprising, as lovastatin is used in the cell-cycle synchronization protocols [92] and as a pharmacological tool for controlling the growth of neoplastic cells both in vitro and in vivo [23,93,94]. Furthermore, lovastatin is commercially available as an inhibitor of the cell cycle in the G1 and G2/M phases (Sigma, USA). The G1 block has been attributed to the inhibition of either cytokinesis or cell spreading following cytokinesis [95]. Several authors have also noted the retardation or arrest of the cell cycle at the G2/M transition [93,94,96]. However, the mechanism of the cell-cycle arrest by statins is not exactly clear. Despite a piece of evidence that lovastatin suppresses cell proliferation through inhibition of proteasome-mediated degradation of p21 and p27 [97], it was concluded that lovastatin neither synchronizes cells nor arrests the cells in the G1 phase of the division cycle [98].

To explain the reported results, the distinct impact of statins on gene expression profiles in MiaPaCa-2 cells should be related to their inhibitory activity interfering with the mevalonate pathway. Quantum biochemistry computations indicated some variations among the attractive forces of four tested statins (atorvastatin, rosuvastatin, simvastatin, and fluvastatin) and the HMG-CoA reductase binding site. The highest binding energies was determined for atorvastatin followed by rosuvastatin, while the lowest were found for simvastatin and fluvastatin; i.e., binding energies of 320, 310, 290, and 290 kcal·mol^-1^, respectively [99]. However, in this study, cerivastatin, lovastatin, and pitavastatin were not included in the calculations. In another study, the crystal structures of the catalytic moiety of HMG-CoA reductase in a complex with six statins documented van der Waals interactions of the rigid hydrophobic moieties of the statins through a shallow nonpolar binding pocket and a part of the binding surface for CoA [100]. These interactions prevented the binding of the substrate HMG-CoA to the active site of the enzyme. No dramatic differences were found among the numbers of binding interactions among the statins evaluated, namely: compactin, simvastatin, fluvastatin, cerivastatin, atorvastatin, and rosuvastatin. Atorvastatin, simvastatin, lovastatin, fluvastatin, and cerivastatin are relatively lipophilic and are metabolized by the cytochrome P450 system. The other lipophilic compound pitavastatin is metabolized poorly via this pathway. Very limited P450-mediated metabolization was reported also for hydrophilic pravastatin and rosuvastatin, which were only nonsignificantly metabolized. Interestingly, high systemic bioavailability was reported for both cerivastatin and pitavastatin (60% [101] and 80% [102], respectively), which could explain their large impact on changes in gene expression. Except for pravastatin, all the other statins were efficiently bound to plasma proteins. However, the unbound pravastatin was poorly distributed in tissues due to its high hydrophilic nature [103].

Further, the distinct efficacy of individual statins on both antiproliferative activity and changes in gene expression could be also correlated with the statin levels inside cells. This correlation was the strongest for the least efficient statins (rosuvastatin and pravastatin); whereas for the most bioavailable statins (in particular lovastatin), this correlation was not so strong [30]. Thus, the statin effects on whole gene expression correlated with their bioavailability, as well as the impact on cell viability, only to a limited extent. This hypothesis held for cerivastatin; however, not so for pitavastatin and lovastatin, which led us to the conclusion that other crucial factors played an important role in the differences of statin effects on pancreatic cancer cell proliferation.

In summary, differences in the efficacy of individual statins are known depending on their structure, concentration, duration of action, or microenvironment. Although tumor cells exhibit many identical properties, the effect of statins depends also on the cell type [104]. The antitumor effect of statins is not only a function of the mechanism of their action, but also of how they are metabolized. In general, healthy cells are generally more resistant to statins than tumor cells [105]. The use of statins in the treatment of cancer as monotherapeutics is not effective enough, but their use in combination with other therapeutic approaches would significantly increase the effectiveness of cancer treatments and patient survival [106,107].

## Figures and Tables

**Table 1:** Genes involved in the metabolism of lipids and steroids that were affected by statin treatment of MiaPaCa-2 cells. Numeric columns display fold changes of the gene expression between cells treated by individual statins at 12 µM concentration for 24 h and untreated cells. Cer.—cerivastatin; Pit.—pitavastatin; Sim.—simvastatin; Flu.—fluvastatin; Ato.—atorvastatin; Lov.—lovastatin. Pravastatin and rosuvastatin did not induce any significant change in MiaPaCa-2 cell gene expression.

Gene Symbol	Ref. ID	Product Name	Cer.	Pit.	Sim.	Flu.	Ato.	Lov.
			Fold Change					
*HMGCS1*	NM_002130	HMG-CoA synthase (EC 2.3.3.10)	5.67	2.99	4.13	2.96	2.90	2.80
*HMGCR*	NM_000859	HMG-CoA reductase (EC 1.1.1.34)	3.91	3.16	3.04	2.66	2.35	2.08
*MVD*	NM_002461	Mevalonate pyrophosphate decarboxylase (EC 4.1.1.33)	3.66	2.11	2.32	2.01	-	-
*PPAP2A*	NM_003711	Phosphatidic acid phosphatase 2a (EC 3.1.3.4)	3.41	2.78	2.15	-	-	-
*AGPAT2*	NM_006412	1-acyl-glycerol-phosphate acyltransferase 2 (EC 2.3.1.51)	2.63	2.28	-	-	-	-

**Table 2:** Epigenetic regulator genes that were affected by statin treatment of MiaPaCa-2 cells. Numeric columns display fold changes of the gene expression between cells treated by individual statins at 12 µM concentration for 24 h and untreated cells. Cer.—cerivastatin; Pit.—pitavastatin; Sim.—simvastatin; Flu.—fluvastatin. Atorvastatin, lovastatin, pravastatin, and rosuvastatin did not induce any significant change in MiaPaCa-2 cells’ gene expression.

Gene Symbol	Ref. ID	Product Name	Cer.	Pit.	Sim.	Flu.
			Fold Change			
*HIST1H4C*	NM_003542.3	Histone H4	0.41	0.34	0.49	-
*HIST1H2BF*	NM_003522.3	Histone H2B type 1-K	0.33	0.41	0.43	0.49
*HIST2H3C*	NM_021059.2	Histone H3C type 2	-	0.50	-	-
*H2AFX*	NM_002105.2	Histone H2A family member X	-	0.43	-	-
*HIST2H2BE*	NM_003528.2	Histone H2B type 2-E	4.32	4.33	2.05	-
*HIST1H2BD*	NM_138720.1	Histone H2B type 1-D	4.15	4.55	2.01	-
*HIST1H2BK*	NM_080593.1	Histone H2B type 1-K	2.10	2.67	-	-
*HIST3H2A*	NM_033445.2	Histone H2A type 3	-	2.02	-	-

**Table 3:** Genes involved in posttranslational regulation affected by statin treatment of MiaPaCa-2 cells. Numeric columns display fold changes of the gene expression between cells treated by individual statins at 12 µM concentration for 24 h and untreated cells. Cer.—cerivastatin; Pit.—pitavastatin; Sim.—simvastatin; Flu.—fluvastatin; Ato.—atorvastatin; Lov.—lovastatin. Pravastatin and rosuvastatin did not induce any significant change in MiaPaCa-2 cell gene expression.

Gene Symbol	Ref. ID	Product Name	Cer.	Pit.	Sim.	Flu.	Ato.	Lov.
			Fold Change					
*RHOB*	NM_004040.2	Ras homolog gene family, member B	12.48	11.90	10.81	7.11	5.59	4.92
*RASL10A*	NM_001007279.1	RAS-like, family 10, member A	4.14	3.58	2.65	2.17	2.01	-
*KRAS*	NM_033360.2	Kirsten rat sarcoma viral oncogene homolog	3.23	2.64	2.58	2.00	-	-
*RAB40B*	NM_006822.1	Member RAS oncogene family	3.14	2.89	2.16	-	-	-
*RRAS*	NM_006270.3	Related RAS viral (r-ras) oncogene homolog	2.58	2.53	2.04	-	-	-
*RAB5B*	NM_002868.2	RAS oncogene family member	2.46	2.04	-	-	-	-
*RAB6B*	NM_016577.3	RAS oncogene family member	2.32	2.31	-	-	-	-
*RHOA*	NM_001664.2	Ras homolog gene family, member A	2.21	2.40	2.03	-	-	-
*ARHGEF3*	NM_019555.1	Rho guanine nucleotide exchange factor (GEF) 3	2.21	2.09	-	-	-	-
*RHOQ*	NM_012249.3	Ras homolog gene family, member Q	2.17	-	-	-	-	-
*RRAGC*	NM_022157.2	Ras-related GTP binding C	2.06	-	-	-	-	-
*RAB38*	NM_022337.1	Member RAS oncogene family	0.41	-	-	-	-	-
*ARHGAP19*	NM_032900.4	Rho GTPase activating protein 19	0.47	0.38	-	-	-	-

**Table 4:** Genes involved in the cell cycle and DNA replication affected by statin treatment of MiaPaCa-2 cells. Numeric columns display fold changes of the gene expression between cells treated by individual statins at 12 µM concentration for 24 h and untreated cells. Cer.—cerivastatin; Pit.—pitavastatin; Sim.—simvastatin; Flu.—fluvastatin; Ato.—atorvastatin; Lov.—lovastatin. Pravastatin and rosuvastatin did not induce any significant change in MiaPaCa-2 cell gene expression.

Gene Symbol	Ref. ID	Product Name	Cer.	Pit.	Sim.	Flu.	Ato.	Lov.
			Fold Change					
*CDKN1A*	NM_000389.2	Cyclin-dependent kinase inhibitor 1A (p21, Cip1)	3.64	4.21	2.74	2.59	2.33	2.40
*SFN*	NM_006142.3	Stratifin	2.40	2.57	-	-	-	-
*CDKN1C*	NM_057735.1	Cyclin-dependent kinase inhibitor 1C (p57, Kip2)	-	2.37	-	-	-	-
*CCNE2*	NM_057735.1	Cyclin E2	0.30	0.29	-	-	-	-
*CDC25A*	NM_001789.2	Cell division cycle 25 homolog A	0.30	0.33	-	-	-	-
*ORC1L*	NM_004153.2	Origin recognition complex, subunit 1-like	0.30	0.22	-	-	-	-
*ORC6L*	NM_014321.2	Origin recognition complex, subunit 6 like	0.31	0.30	-	-	-	-
*SKP2*	NM_005983.2	S-phase kinase-associated protein 2 (p45)	0.34	0.37	-	-	-	-
*MCM7*	NM_005916.3	Minichromosome maintenance complex component 7	0.38	0.45	-	-	-	-
*E2F2*	NM_004091.2	E2F transcription factor 2	0.39	0.40	-	-	-	-
*CDC45L*	NM_003504.3	CDC45 cell division cycle 45-like	0.39	0.27	-	-	-	-
*MCM2*	NM_004526.2	Minichromosome maintenance complex component 2	0.40	0.30	-	-	-	-
*MCM3*	NM_002388.3	Minichromosome maintenance complex component 3	0.41	0.33	-	-	-	-
*CDC6*	NM_001254.3	Cell division cycle 6 homolog	0.41	0.47	-	-	-	-
*CDC2*	NM_001786.2	Cell division cycle 2, G1 to S and G2 to M	0.41	0.32	-	-	-	-
*CCND1*	NM_053056.2	Cyclin D1	0.43	-	-	-	-	-
*MCM4*	NM_005914.2	Minichromosome maintenance complex component 4	0.43	0.36	-	-	-	-
*MCM5*	NM_006739.3	Minichromosome maintenance complex component 5	0.45	0.35	-	-	-	-
*PCNA*	NM_182649.1	Proliferating cell nuclear antigen (PCNA), transcript variant 2	0.46	0.45	-	-	-	-
*CDK2*	NM_001798.2	Cyclin-dependent kinase 2	0.48	0.44	-	-	-	-
*PKMYT1*	NM_182687.1	Protein kinase, membrane associated tyrosine/threonine 1	0.49	0.30	-	-	-	-
*MAD2L1*	NM_002358.2	MAD2 mitotic arrest deficient-like 1	0.49	0.36	-	-	-	-
*CCNA2*	NM_001237.2	Cyclin A2	-	0.31	-	-	-	-
*TTK*	NM_003318.3	TTK protein kinase	-	0.41	-	-	-	-
*CDC20*	NM_001255.2	Cell division cycle 20 homolog	-	0.46	-	-	-	-
*CDC25C*	NM_001790.3	Cell division cycle 25 homolog C	-	0.46	-	-	-	-
*PLK1*	NM_005030.3	Polo-like kinase 1	-	0.47	-	-	-	-
*BUB1*	NM_004336.2	BUB1 budding uninhibited by benzimidazoles 1 homolog	-	0.48	-	-	-	-

**Table 5:** Genes involved in cell death affected by statin treatment of MiaPaCa-2 cells. Numeric columns display fold changes of the gene expression between cells treated by individual statins at 12 µM concentration for 24 h and untreated cells. Cer.—cerivastatin; Pit.—pitavastatin; Sim.—simvastatin; Flu.—fluvastatin; Ato.—atorvastatin; Lov.—lovastatin. Pravastatin and rosuvastatin did not induce any significant change in MiaPaCa-2 cell gene expression.

Gene Symbol	Ref. ID	Product Name	Cer.	Pit.	Sim.	Flu.	Ato.	Lov.
			Fold Change					
*TNFRSF10D*	NM_003840.3	Tumor necrosis factor receptor superfamily, member 10d	3.71	3.17	4.68	3.06	2.76	2.60
*SLC6A12*	NM_003044.2	Solute carrier family 6 (betaine/GABA), member 12	2.45	2.16	2.84	2.45	-	-
*DRAM*	NM_018370.2	Damage-regulated autophagy modulator	2.18	2.41	-	-	-	-
*CASP9*	NM_032996.1	Caspase 9, apoptosis-related cysteine peptidase	2.16	-	-	-	-	-
*GABARAPL1*	NM_031412.2	GABA(A) receptor-associated protein like 1	2.13	3.76	2.49	2.13	-	-
*ATG2A*	NM_015104.1	ATG2 autophagy related 2 homolog A	2.08	-	-	-	-	-
*TNFAIP1*	NM_021137.3	Tumor necrosis factor, alpha-induced protein 1	2.01	-	-	-	-	-
*CARD10*	NM_014550.3	Caspase recruitment domain family, member 10	0.43	0.49	-	-	-	-
*TNFRSF6B*	NM_032945.2	Tumor necrosis factor receptor superfamily, member 6b	0.48	-	-	-	-	-

**Table 6:** Genes involved in cytoskeleton maintenance affected by statin treatment of MiaPaCa-2 cells. Numeric columns display fold changes of the gene expression between cells treated by individual statins at 12 µM concentration for 24 h and untreated cells. Cer.—cerivastatin; Pit.—pitavastatin; Sim.—simvastatin; Flu.—fluvastatin; Ato.—atorvastatin; Lov.—lovastatin. Pravastatin and rosuvastatin did not induce any significant change in MiaPaCa-2 cell gene expression.

Gene Symbol	Ref. ID	Product Name	Cer.	Pit.	Sim.	Flu.	Ato.	Lov.
			Fold Change					
*LOC400578*	XR_017543.1	Similar to keratin, type I cytoskeletal 14 (Cytokeratin-14)	15.80	12.21	5.44	3.62	3.32	2.59
*KRT16*	NM_005557.2	Keratin 16	13.85	12.21	4.60	3.52	3.28	2.23
*MGC102966*	XR_015970.1	Similar to keratin, type I cytoskeletal 16 (Cytokeratin-16)	13.54	11.95	4.84	3.53	3.18	2.31
*KRT15*	NM_002275.2	Homo sapiens keratin 15	5.97	5.86	3.85	3.43	3.02	3.25
*KLHL24*	NM_017644.3	Kelch-like 24	5.78	4.56	2.89	2.21	2.21	-
*JUP*	NM_002230.1	Junction plakoglobin	5.33	4.09	2.78	2.51	2.00	-
*TUBB1*	NM_030773.2	Tubulin, beta 1	4.02	-	-	2.23	-	-
*KRT13*	NM_002274.3	Keratin 13	3.16	4.35	3.38	2.84	2.68	3.05
*KRT19*	NM_002276.3	Keratin 19	3.13	2.88	2.50	2.20	2.11	-
*GSN*	NM_198252.2	Gelsolin (amyloidosis, Finnish type)	2.97	2.32	2.45	2.00	-	-
*KIF1A*	NM_004321.4	Kinesin family member 1A	2.73	3.05	2.26	-	-	-
*TNNT1*	NM_003283.3	Troponin T, slow skeletal muscle	2.70	3.57	2.10	-	-	-
*ITGB4*	NM_001005619.1	Homo sapiens integrin, beta 4	2.45	2.49	2.18	2.03	-	2.20
*VIL2*	NM_003379.3	Villin 2 (ezrin)	2.16	2.18	2.01	-	-	-
*NAV1*	NM_020443.2	Neuron navigator 1	2.14	2.38	2.13	-	-	-
*GSN*	NM_198252.2	Gelsolin (amyloidosis, Finnish type)	-	-	-	-	-	-
*CDH10*	NM_006727.2	Cadherin 10, type 2 (T2-cadherin)	0.31	0.28	0.33	0.48	0.45	-
*SYNM*	NM_015286.5	Synemin, intermediate filament protein	0.48	0.50	-	-	-	-

## Data Availability

The gene microarray data have been deposited in the ArrayExpress database (accession number E-MTAB-3263).

## Supplementary Materials

The following are available online: Table S1: Legend for figures from STRING database.
